# Direct effects of caffeine on osteoblastic cells metabolism: the possible causal effect of caffeine on the formation of osteoporosis

**DOI:** 10.1186/1749-799X-1-7

**Published:** 2006-10-07

**Authors:** Yang-Hwei Tsuang, Jui-Sheng Sun, Li-Ting Chen, Samuel Chung-Kai Sun, San-Chi Chen

**Affiliations:** 1 Department of Orthopedic Surgery, Taipei City Hospital, Taipei, Taiwan, ROC; 2 Institute of Rehabilitation Science and Technology, National Yang-Ming University, Taipei, Taiwan, ROC; 3 HealthBanks Biotechnology Cooperation Limited, Taipei, Taiwan, ROC; 4 Department of Biochemistry, Queen's University, Kingston, Ontario, Canada; 5 Department of Orthopedic Surgery, Cathay General Hospital, Taipei, Taiwan, ROC

## Abstract

**Background:**

Caffeine consumption has been reported to decrease bone mineral density (BMD), increase the risk of hip fracture, and negatively influence calcium retention. In this study, we investigated the influence of caffeine on the osteoblasts behaviour.

**Method:**

Osteoblasts derived from newborn Wistar-rat calvaria was used in this study. The effects of various concentrations of caffeine on bone cell activities were evaluated by using MTT assay. Alkaline phosphatase (ALP) staining, von Kossa staining and biochemical parameters including ALP, lactate dehydrogenase (LDH), prostaglandin E_2 _(PGE_2_) and total protein were performed at day 1, 3, and 7. DNA degradation analysis under the caffeine influence was also performed.

**Results and discussion:**

The results showed that the viability of the osteoblasts, the formation of ALP positive staining colonies and mineralization nodules formation in the osteoblasts cultures decreased significantly in the presence of 10 mM caffeine. The intracellular LDH, ALP and PGE_2 _content decreased significantly, the LDH and PGE_2 _secreted into the medium increased significantly. The activation of an irreversible commitment to cell death by caffeine was clearly demonstrated by DNA ladder staining.

**Conclusion:**

In summary, our results suggest that caffeine has potential deleterious effect on the osteoblasts viability, which may enhance the rate of osteoblasts apoptosis.

## Background

Caffeine and the related methyl xanthines are widely distributed in plants throughout the world. All stable indigenous cultures having access to these plant products have developed drinks containing these stimulants. Thus caffeine is probably the most commonly consumed pharmacologically active compound in the world, certainly in Europe and North America. Caffeine-containing beverage consumption has been reported to be associated with reduced bone mass and increased fracture risk in some observational studies. In 1982, Heaney and Recker's publication first showed a negative effect of caffeine on the calcium economy [1]. Shortly thereafter, Massey and colleagues [2] showed that a caffeine-induced diuresis increased urinary calcium loss acutely. Later controlled human physiological balance studies show a clear but only a very small depressant effect of caffeine on intestinal calcium absorption, and no effect on total 24-h urinary calcium excretion [3].

The role of caffeine as a risk factor for bone loss is still controversial. Caffeine consumption has been reported to decrease bone mineral density (BMD) [4], increase the risk of hip fracture [5], and negatively influence calcium retention [6,7]. However, most of the studies reported no overall association between caffeine intake and BMD, fracture rate, or calcium metabolism [8-14]. In a longitudinal study about the interaction between caffeine intake, vitamin D receptor (VDR) polymorphism, and bone mineral density (BMD), Rapuri et al. demonstrated that if the intakes of caffeine in amounts more than 300 mg/d (approximately 514 g, or 18 oz, brewed coffee) accelerated bone loss at the spine in elderly postmenopausal women [15].

There are four probable ways an agent may increase the fracture risk and/or skeletal fragility of an elder people [16]: (1) an interference with the bone remodeling process designed to repair micro-fracture and/or fatigue damage in bone structures; (2) lowered daily activity followed by a decrease in bone tissue mass and change in the optimal orientation of bony trabeculae; (3) an interference with postural reflexes and/or an increase in fall frequency; and (4) a reduction of body fat over bony prominences during the aging process. On these grounds, caffeine may lead to substantial modifications of the probable contributor to the osteoporosis disease. Generally, the first two mechanism are still inadequately explored for bone and its importance for osteoporotic fractures remains undefined. Also, there are no recognized data relating caffeine to the third and forth mechanisms. In this study, we investigated the influence of caffeine on in vitro osteoblasts metabolism. The biocompatibility has been evaluated by means of cytotoxicity and cyto-compatibility tests. Cell proliferation as well as the expression of some biochemical parameters of osteoblastic phenotypes have been monitored, the effect of caffeine on the osteoblasts viability was also evaluated.

## Methods

### Preparation of caffeine solutions

The powder of caffeine (Sigma, St. Louis, MO, USA) were purchased and diluted in phosphate buffered solution (Sigma, St. Louis, MO, USA). In the first part of this study, the effects of various concentrations of caffeine on bone cell activities were evaluated by using MTT assay as described below. Seven different concentrations (100, 50, 10, 5, 1, 0.5, 0.1 mM) were tested for 1 day, 3 days, 7 days and 14 days period.

### Osteoblast cell culture

Sequential digestion of newborn Wistar-rat calvaria was performed by using modification of the methods previously described [17]. To subculture, the cells were washed with sterile PBS followed by treatment with 1:1 mixture of 0.03% collagenase and 0.05% trypsin (Sigma, St. Louis, MO, USA) for 20 minutes at 37°C in 5% C0_2_. The resulting cell suspension was then passed and centrifuged at 1500 rpm for five minutes to pellet the cells. The supernatant was removed and the pellet re-suspended in α-minimal essential media (α-MEM; Sigma, St. Louis, MO, USA) as described below. Unambiguous identification of cell populations as osteoblasts is complex and none of the parameters used for defining osteoblasts-like cells are unique to this cell types. The presence of alkaline phosphatase, an early marker of osteoblasts [18], is used to assess the osteoblastic character of the isolated cells [19].

### Colorimetric assay for cell viability [20]

The mitochondria activity of the bone cells after exposure to various concentrations of caffeine was determined by colorimetric assay which detects the conversion of 3-(4,5-dimethylthiazolyl-2)-2,5-diphenyltetrazolium bromide (MTT, Sigma Co., St. Louis, MO, USA) to formazan. For the assay, 2.5 × 10^4 ^cells per well were incubated (5% CO_2_, 37°C) in the presence of various concentration of caffeine. After various time intervals the supernatant was removed, 100 μl per well of MTT solution (1 mg/ml in test medium) was added and the wells were incubated at 37°C for 4 h to allow the formation of formazan crystal. All crystals were dissolved, the plates were read on Micro Elisa reader (Emax Science Corp., Sunnyvale, California, USA) at wavelength of 570 nm against a reference wavelength of 690 nm.

### Osteoblast differentiation

Osteoblasts cultured in the media in the presence of dexamethasone have been shown to be capable of synthesizing and mineralizing an extracellular matrix and to form alkaline phosphatase in vitro [21]. To test the differentiation of osteoblasts, a concentration of 1 × 10^5 ^cells/100 μl was added to 35 mm wells of a 6-well plate. The osteoblasts were incubated at 37°C in 5% C0_2 _for 48 hours. After 48 hours, the media were changed and the cells were incubated in α-MEM supplemented with 10% fetal calf serum (FCS; Gibco BRL, Rockville, MD, USA), antibiotics (gentamicin 50 μg/ml, penicillin G 100 μg/ml [Gibco BRL, Rockville, MD, USA]), L-ascorbic acid (50 μg/ml Gibco BRL, Rockville, MD, USA), supplemented with 5 mM β-glycerophosphate (Sigma, St. Louis, MO, USA) and 10^-8 ^M dexamethasone (Sigma, St. Louis, MO, USA). The day of changing specific medium was day zero. From day zero of culture, 10 mM caffeine solution was added. The medium was changed every 3–4 days; alkaline phosphatase (ALP) staining, von Kossa stain for mineralized nodules and biochemical parameters including alkaline phosphatase, lactate dehydrogenase, prostaglandin E_2 _and total protein were performed at day 1, 3, and 7.

### Alkaline Phosphatase (ALP) staining

After fixing the cells, the dishes were incubated for 30 minutes in TRIS Buffer (0.2 M, pH 8.3) with AS-MX phosphate (Sigma, St. Louis, MO, USA) as a substrate and Fast Blue (Sigma, St. Louis, MO, USA) as a stain. The ALP positive cells stained blue/purple. For each experiment, a minimum of three dishes was counted and the experiments were repeated three times.

### The von-Kossa staining on mineralized nodules formation

Mineralization of the nodules in the cultures was assessed using von-Kossa stain. The matrix was washed with PBS, and cultures were treated with 5% silver nitrate solution 100 μL/well in the dark at 37°C for 30 minutes. The excess silver nitrate solution was then completely washed away using double-distilled H_2_O and the culture plate was exposed to sodium carbonate/formaldehyde solution for few minutes to develop color. The von Kossa-stained areas were viewed by light microscopy. For each experiment, a minimum of three dishes was counted and the experiments were repeated three times.

### Analysis of alkaline phosphatase, lactate dehydrogenase, prostaglandin E_2 _and total protein in culture medium

Alkaline phosphatase (ALP), lactate dehydrogenase (LDH) activities and total protein released from the cells into the medium were measured with a commercially available assay kit (ALP: Procedure no. ALP-10; Procedure no. 435, LDH: Procedure no. 228-UV, LDL-10, TP: Procedure no.690-A, Sigma Co., St. Louis, MO, USA). The production of prostaglandin E_2 _(PGE_2_) in culture medium was also analyzed with a commercially available assay kit (Cayman Chemical Company, MI, USA).

### Analysis of intracellular ALP, LDH, PGE_2 _and total protein

At the end of the experimental period, ALP, LDH, PGE_2 _and TP activities were determined following lysis of the cells with the detergent Triton X-100 (Sigma, St Louis, MO, USA). Intracellular ALP, LDH, PGE_2 _and TP values were determined as the methods described for the measurements of culture media.

### Statistical analysis

All data were expressed as mean ± standard deviation and were analyzed by analysis of variance. Statistical significance was determined by Bonferroni's t-test. Probability values less than 0.05 were considered significant.

### DNA degradation analysis

For the DNA fragmentation, a concentration of 1 × 10^6 ^cells/100 μl was added to 90 mm disc. From day one of culture, six different concentrations of caffeine solution (0, 0.5, 1.0, 2.5, 5.0, 10.0 nM) were added. The medium was changed every 3–4 days, the DNA fragmentation analyses were performed at day 1, 3, and 7. For the test, floating and adherent cells from each culture condition were combined, centrifuged, pelleted at 400 × *g *for 5 min, and washed twice with PBS. The pellet was resuspended in 0.2 ml lysis buffer [100 mM NaCl, 10 mM Tris (pH 8.0), 1 mM EDTA, 0.5% sodium dodecyl sulfate, 0.20 mg/ml proteinase K, 200 μg/ml ribonuclease A]. The cell lysates were then incubated at 37°C for 2 h. The genomic DNA was extracted by two separations, with phenol/chloroform and then with chloroform only. The DNA pellet was then washed in 70% ethanol and resuspended in 1 mM EDTA, 10 mM Tris-HCl (pH 8.0) at a final concentration of 20 μg/ml. The DNA fragmentation analysis was performed using a 1.5% agarose gel in 1 mM EDTA, 40 mM Tris acetate (pH 7.6) to visualize the laddering of the samples.

## Results

### Quantitative analysis of osteoblast cell counts

**Figure 1:**
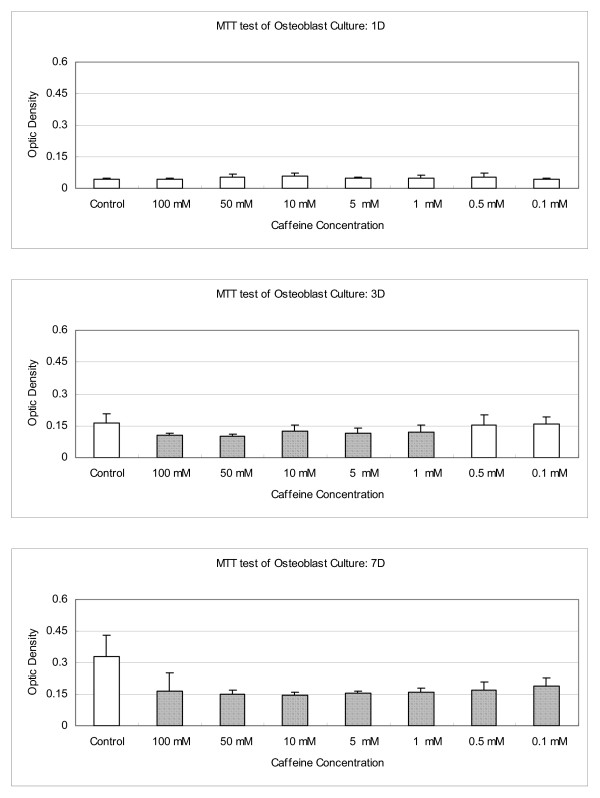
**The effect of caffeine on osteoblast cells viability measured by MTT assay**. When osteoblast cells cultured with caffeine for one day, there was no statistically significant change in the formation of formazan; while in the 100 mM to 1 mM concentration of caffeine, the formation of formazan was significantly decreased in the third day's culture. At the 7^th ^day's culture, decreased osteoblasts activities were observed in the presence of various concentrations of caffeine. We selected the 10 mM concentration of caffeine for the further biochemical study because the osteoblasts showed the highest activities during the 3^rd ^and 7^th ^testing period. (Shaded bars mean significant differences to that control: P < 0.05).

Figure 1 shows the effect of various concentrations of caffeine on osteoblast cells viability measured by MTT assay. When osteoblast cells cultured with caffeine for one day, there was no statistically significant change in the formation of formazan; while in the 100 mM to 1 mM concentration of caffeine, the formation of formazan was significantly decreased in the third day's culture (Fig. 1). At the 7^th ^day's culture, decreased osteoblasts activities were observed in the presence of various concentrations of caffeine. We selected the 10 mM concentration of caffeine for the further biochemical study because the osteoblasts showed the highest activities during the 3^rd ^and 7^th ^testing period (Fig. 1).

### Alkaline phosphatase staining and mineralized nodules formation

**Figure 2:**
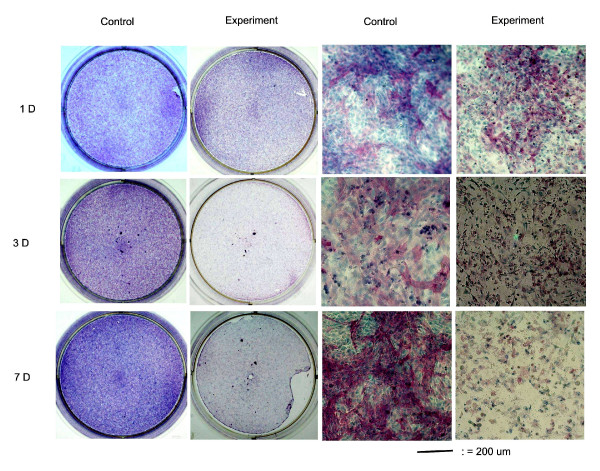
**Alkaline Phosphatase Staining**. The alkaline phosphatase positive staining colonies first appeared at the 1^st ^day's culture of control groups, and then progressively increased as the culture period passed, and attained a significant degree at the 7^th ^day's culture. When osteoblasts cultured with 10 mM concentration of caffeine, the appearance of alkaline phosphatase staining was likely affected a lot. In the presence of 10 mM caffeine, the viability of osteoblasts was decreased and the residual cells lost their reaction to alkaline phosphatase staining.

**Figure 3:**
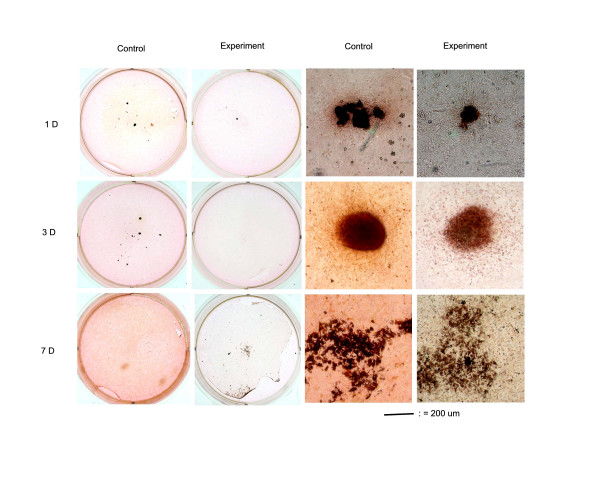
**Von-Kossa Staining and Mineralized Nodules Formation**. At 3 hours after differentiation medium, little Von-Kossa positive staining colony was found in the culture. The Von-Kossa positive staining colonies first appeared at the 1^st ^day's culture of control groups, and then progressively increased as the culture period passed, and attained a significant degree at the 7^th ^day's culture. When osteoblasts cultured with 10 mM concentration of caffeine, the appearance of Von-Kossa staining was decreased and the residual cells lost their reaction to Von-Kossa staining.

In control samples, the osteoblasts differentiated as the cultured period increased. At 3 hours, little alkaline phosphatase positive staining colony was found in the culture. The alkaline phosphatase positive staining colonies first appeared at the 1^st ^day's culture of control groups, and then progressively increased as the culture period passed, and attained a significant degree at the 7^th ^day's culture (Fig. 2). When osteoblasts cultured with 10 mM caffeine, the appearance of alkaline phosphatase staining was likely affected (Fig. 2). In the presence of 10 mM caffeine, the viability of osteoblasts was decreased and the residual cells lost their reaction to ALP stain and similar results were observed on the von-Kossa staining (Fig. 3). The formation of ALP positive staining colonies and mineralization nodules formation in the osteoblast cultures were significantly affected by caffeine.

### Alkaline phosphatase (ALP), Lactate dehydrogenase (LDH), Prostaglandin E_2 _(PGE_2_) and Total protein (TP)

**Figure 4:**
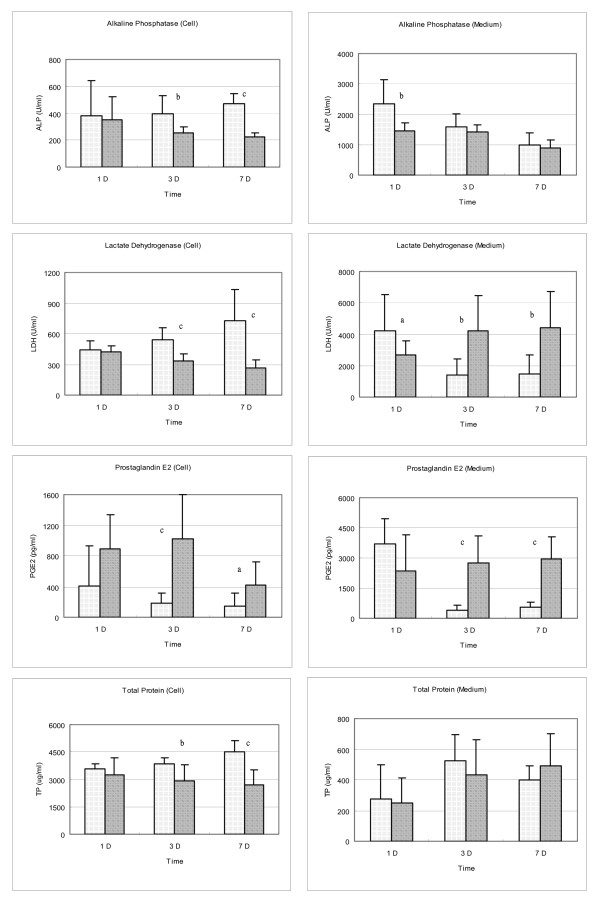
**Effects of caffeine on the osteoblasts: Changes in biochemical parameters**. For the bone cells culture, intracellular total protein, alkaline phosphatase (ALP) and lactate dehydrogenase (LDH) synthesis were increased gradually while the prostaglandin E_2 _(PGE_2_) synthesis decreased during the 7 days' culture period. At the same time, ALP, LDH, and PGE_2 _secretion into medium decreased, while the total protein in the culture medium was relatively stationary. After adding 10 mM caffeine to the osteoblasts cell culture for 3 to 7 days, the intracellular ALP content decreased significantly, while the ALP secreted into medium was relatively preserved. The intracellular LDH decreased significantly and the LDH in the medium increased significantly at the presence of 10 mM caffeine for 3 to 7 days. Both the intracellular PGE_2 _and the PGE_2 _secreted into medium decreased significantly at the 3^rd ^and 7^th ^day's culture. At the same time, total protein contents were relatively preserved.

For the bone cells culture, intracellular total protein, ALP and LDH synthesis were increased gradually while the PGE_2 _synthesis decreased during the7 days' culture period (Fig. 4). At the same time, ALP, LDH, and PGE_2 _secretion into medium decreased, while the total protein in the culture medium was relatively constant (Fig. 4). After adding 10 mM caffeine to the osteoblasts cell culture for 3 to 7 days, the intracellular ALP content decreased significantly, while the ALP secreted into medium was relatively preserved (Fig. 4). The intracellular LDH decreased significantly and the LDH in the medium increased significantly at the presence of 10 mM caffeine for 3 to 7 days (Fig. 4). Both the intracellular PGE_2 _and the PGE_2 _secreted into medium decreased significantly at the 3^rd ^and 7^th ^day's culture (Fig. 4). At the same time, total protein contents were relatively preserved (Fig. 4).

### DNA degradation analysis

**Figure 5:**
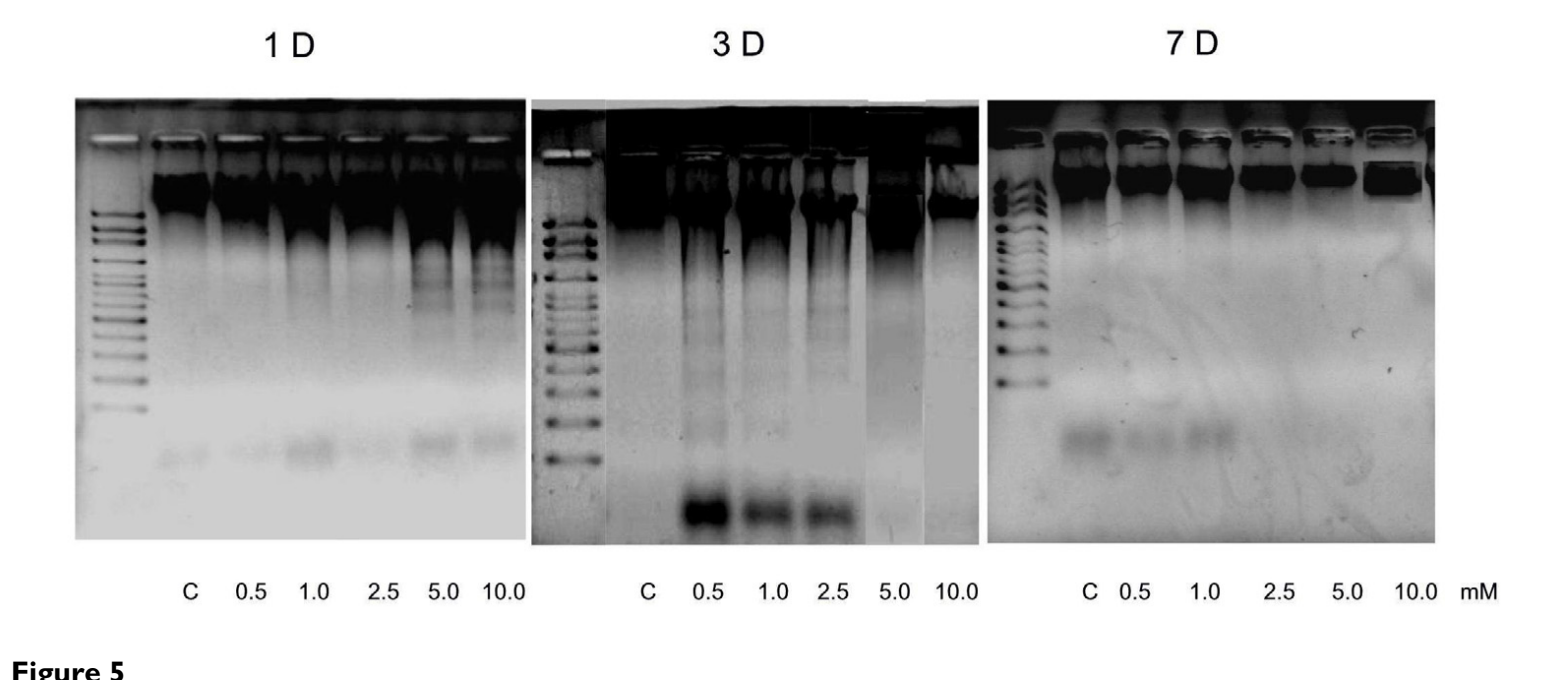
**Osteobalsts DNA degradation induced by caffeine**. Activation of an irreversible commitment to cell death by caffeine was clearly demonstrated in the DNA fragmentation analysis. The formation of DNA fragments of was easily observed when osteoblasts cultured with caffeine. Electrophoresis of genomic DNA from osteoblast cells that were exposed to 5.0 and 10 nM caffein showed the characteristic laddering pattern (in the size of 500 – 1000 bp) that led to cell death in the first day's culture; while in the concentrations of 0.5, 1.0 or 2.5 nM caffeine, the appearance of DAN fragmentation appeared at the 3^rd ^day's culture with the characteristic laddering pattern in the size of 200 – 1000 bp.

Activation of an irreversible commitment to cell death by caffeine was clearly demonstrated in the DNA fragmentation analysis. The formation of DNA fragments was easily observed when osteoblasts cultured with caffeine. Electrophoresis of genomic DNA from osteoblasts that were exposed to 5.0 and 10 nM caffeine showed the characteristic laddering pattern (in the size of 500 – 1000 bp) that led to cell death in the first day's culture; while in the concentrations of 0.5, 1.0 or 2.5 nM caffeine, the appearance of DAN fragmentation appeared at the 3^rd ^day's culture with the characteristic laddering pattern in the size of 200 – 1000 bp (Fig. 5).

## Discussion

Coffee is one of the most widely consumed psychoactive beverages throughout the world. Many investigators have demonstrated that caffeine, one of the main constituents of coffee, has a variety of pharmacological and cellular responses in the biological systems [22]. These include stimulation of the central nervous system and cardiac muscle, increased urinary output, and relaxation of smooth muscle [23]. The effects of coffee on bone metabolism are still controversial, although several studies have suggested that caffeine and/or heavy coffee consumption are associated with a significant increase in risk of fracture, osteoporosis, and periodontal disease [24,25].

In fact, several epidemiological studies have reported the influence of caffeine on osteoporosis, but the effects of coffee on bone metabolism remain controversial [26,27]. Hypotheses to explain these associations have centered on the caffeine content of coffee [26]. In fact, caffeine has a variety of pharmacological actions and cellular responses in bone metabolism, resulting in increased urinary calcium excretion and in vitro inhibition on the proliferation of osteoblast-like cells [25]. In this study, we found that when osteoblasts cultured with 100 mM to 1 mM concentration of caffeine, the formation of formazan was significantly decreased in the third day's culture. This deleterious effect was even more obvious at the 7^th ^day's culture (Fig. 1). Corresponding to the viability of osteoblasts was decreased significantly in the presence of 10 mM of caffeine, the intracellular LDH and ALP content decreased significantly and the LDH secreted into the medium increased significantly (Fig. 4).

Osteoblasts differentiation is a multistep-events modulated by an integrated cascade of gene expression. These events initially support proliferation, followed by matrix maturation, and mineralization of the bone extracellular matrix [28]. Alkaline phosphatase expression is considered an early differentiation marker of the osteoblasts phenotype, while the von-Kossa stain of mineralized nodules formation represented the end differentiation marker of the osteoblasts. In this study, the differentiation of osteoblasts was induced when β-glycerophosphate and dexamethasone were added into the culture medium [21,29], the degree of differentiation increased as the cultured period increased (Figs. 2 &3) and cultures of osteoblasts also had detectable calcium deposition, as seen on von-Kossa staining by days 7 after the cells reached confluency [30]. In the presence of 10 mM caffeine, the viability of osteoblasts was decreased and the residual cells lost their reaction to ALP staining and von-Kossa staining (Figs. 2 &3). The formation of ALP positive staining colonies and mineralization nodules formation in the osteoblast cultures were significantly affected by caffeine.

Prostaglandins produced by skeletal tissues have complex effects on both catabolic and anabolic activities of bone cells [31]. Prostaglandins (PGs) are local mediators that have diverse effects on bone metabolism. They have been shown to stimulate osteolysis in bone organ cultures [32] and when administrated systemically or locally in vivo, result in increased bone loss [33]. In contrast, PGs directly inhibited the cell activity and bone resorption of isolated osteoclasts [34]. PGs stimulation of osteoclastic activity in intact bone was thought to be mediated indirectly by the action of another cell type in bone, most likely the osteoblasts [35]. In this study, after adding 10 mM caffeine into the culture medium, both the intracellular PGE_2 _content and PGE_2 _secreted into medium increased significantly (Fig. 4); which probably closely correlated with the effects of caffeine on the osteoblasts activities.

Apoptosis, or programmed cell death, is a physiological mode of remodeling tissues during organogenesis and adulthood. The physiological role of programmed cell death (PCD) is aimed at the removal of redundant, misplaced, or damaged cells, or is activated in defense against infected or mutated cells, preventing further proliferation of a pathogen or disease. The process is characterized by morphological changes, including condensation of the nuclear chromatin, DNA fragmentation, cellular shrinkage, and the formation of apoptotic bodies, which are membrane-bound cellular constituents [36]. In animal cells, PCD is often associated with the occurrence of specific biochemical and morphological features such as condensation of the nucleus and cytoplasm, fragmentation of genomic DNA into large (50 to 300 kb) and subsequently small (200 bp) nucleosomal fragments (DNA laddering), and fragmentation of the cell into membrane-confined vesicles (apoptotic bodies) and it is essential to the development and maintenance of multicellular organisms [37]. In this study, the activation of an irreversible commitment to cell death by caffeine was clearly demonstrated when osteoblasts cultured with caffeine (Fig. 5). This fact implied that caffeine may induce osteoblasts apoptosis which then led to decreased bone cells viabilities. The caffeine induced osteoblasts apoptosis probably is one of the major factors in the caffeine-ingestion associated osteoporosis in the clinical medicine. However, this hypothesis is needed to be validated in the further studies.

In summary, our results suggest that caffeine has potential deleterious effect on the osteoblasts viability, which may enhance the rate of osteoblasts apoptosis.
